# Luteinizing hormone is independently associated with high‐sensitive cardiac troponin T elevation in postmenopausal T2DM patients: A cross‐sectional study

**DOI:** 10.1111/1753-0407.70005

**Published:** 2024-10-22

**Authors:** Yahao Wang, Yixuan Li, Chuanfeng Liu, Yangang Wang, Yiming Li

**Affiliations:** ^1^ Department of Endocrinology, Huashan Hospital Fudan University Shanghai China; ^2^ Department of Endocrinology The Affiliated Hospital of Qingdao University Qingdao China

**Keywords:** hs‐cTnT, luteinizing hormone, postmenopause, subclinical myocardial necrosis, type 2 diabetes mellitus

## Abstract

**Background:**

It is known that the risk of ischemic heart disease increases in patients with type 2 diabetes mellitus (T2DM). For female patients, the incidence of heart disease can be even greater after menopause, accompanied by dramatic changes in sex hormones. We investigated the correlations between sex hormones and markers of ischemic heart diseases in postmenopausal females with T2DM patients.

**Methods:**

This cross‐sectional study collected data from 324 hospitalized postmenopausal females with T2DM. Multiple linear regression analyses were conducted to determine the correlations between sex hormones and cardiac markers including high‐sensitive cardiac troponin T (hs‐cTnT) and N‐terminal prohormone of brain natriuretic peptide (NT‐proBNP) levels.

**Results:**

Multiple linear regression analyses revealed that luteinizing hormone (LH) was positively and independently associated with hs‐cTnT concentrations in postmenopausal females with T2DM (*β* = 0.189, *p* = 0.002). Postmenopausal females with T2DM and subclinical myocardial injury had higher LH levels than those without subclinical myocardial injury (29.67 vs. 25.08 mIU/mL, *p* < 0.001). A multivariate logistic regression analysis confirmed an independent and significant association between elevated LH and subclinical myocardial injury in postmenopausal females with T2DM (adjusted odds ratio [OR] = 1.077, 95% confidence interval [CI], 1.033–1.124; *p* < 0.001). As another gonadotropin, the follicle‐stimulating hormone did not show independent correlations with hs‐cTnT or NT‐proBNP (*p* > 0.05). Neither estrogen nor testosterone was correlated with cardiac markers.

**Conclusions:**

Elevated LH levels were positively and independently associated with increased hs‐cTnT levels in postmenopausal women with T2DM. Our findings suggest that LH could serve as a potential marker for assessing the risk of subclinical myocardial injury in postmenopausal females with T2DM.

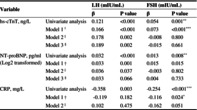

## Graphical abstract

Highlights

- Elevated LH levels in postmenopausal women with T2DM were positively correlated with increased hs‐cTnT.
- LH may emerge as an independent predictor of subclinical myocardial injury risk in postmenopausal T2DM women.

## 1 INTRODUCTION

Type 2 diabetes mellitus (T2DM) is an epidemic disorder.^1^ In postmenopausal women, hormonal changes can lead to imbalances in glycemic metabolism, resulting in impaired insulin secretion and insulin sensitivity, thus increasing the risk of developing T2DM.^2^, ^3^

T2DM is a major risk factor of atherosclerotic cardiovascular disease (ASCVD), and approximately 75% of T2DM patients would die as a consequence of ASCVD.^4^ It is also known that the incidence of ASCVD can be greater after menopause, accompanied by drastic changes in sex hormones.^5^, ^6^ Up to now, the role of sex hormones in the development and progression of ischemic heart diseases in postmenopausal women remains unclear. Most relevant studies have focused on the role of estrogen (estradiol, E2) and testosterone (T) and indicated the protective effect of E2 on the cardiovascular system.^7^ According to a large cohort study, extremely low concentrations of endogenous E2 or extremely high concentrations of T were associated with a high risk of ischemic heart disease.^8^ However, a prospective case–control study suggested that E2 was not independently associated with the risk of coronary heart disease in postmenopausal women.^9^ Hormone therapies based on E2 and progesterone replacement have also shown limited benefit in preventing cardiovascular events in postmenopausal women.^10^, ^11^ Except for changes in E2 and T, menopause is also characterized by a surge in levels of gonadotropins, including luteinizing hormone (LH) and follicle‐stimulating hormone (FSH). However, few reports focus on the role of gonadotropins in the progression of heart diseases in diabetic women. In this study, we aimed to examine the roles of LH and FSH in the manifestation of ischemic heart diseases in postmenopausal female with T2DM.

Among various cardiac markers, high‐sensitive cardiac troponin T (hs‐cTnT) and N‐terminal prohormone of brain natriuretic peptide (NT‐proBNP) are commonly utilized to assess the severity of ischemic heart diseases^12^, ^13^: elevated NT‐proBNP is a well‐known predictor of heart failure, and elevated hs‐cTnT help indicate subclinical myocardial injury.^14^, ^15^, ^16^ Above all, we designed this cross‐sectional study to investigate the relationship between sex hormones and cardiac markers, including hs‐cTnT and NT‐proBNP, in postmenopausal female with T2DM. The associations between hs‐cTnT elevation and several anthropometric/metabolic factors except for sex hormones were also explored.

## 2 MATERIALS AND METHODS

### 2.1 Study population

Participants in this study were hospitalized at the Affiliated Hospital of Qingdao University (Shandong, China). Data were collected from the inpatient database of postmenopausal female with T2DM between 2013 and 2022. The inclusion criteria were as follows: (1) Female T2DM patients; (2) patients at the postmenopausal stage; (3) patients with assay outcomes for both gonadotropins and key cardiac markers, such as hs‐cTnT; (4) patients with complete clinical data; (5) individuals aged between 45 and 85 years. We considered a woman postmenopausal if she confirmed menopause or was ≥60 years old or ≥55 years old with FSH ≥25 mIU/mL. The participants with the age <45 years or >85 years, acute diabetic complications, severe liver or kidney diseases, malignant tumors, history of polycystic ovarian syndrome or pituitary tumor, or autoimmune diseases were excluded. Women who were premenopausal, perimenopausal, or those with unknown menopausal status, or those taking the hormone therapy were not included. We included only postmenopausal women in this study because sex hormone levels and mechanistic pathways might be different between pre‐ and postmenopausal women. The study was performed according to the Declaration of Helsinki and was approved by the ethics committee of our hospital.

### 2.2 Data collection

We analyzed the characteristics of the study population including age, gender, height, weight and hip circumference, smoking and alcohol status, diabetes duration, blood glucose, blood pressure (BP), lipid profiles including low‐density lipoprotein cholesterol (LDL‐C), high‐density lipoprotein cholesterol (HDL‐C), total cholesterol (TC) and triglycerides (TG), microalbuminuria (mAlb), C‐reactive protein (CRP), sex hormones including LH, FSH, T and E2, cardiac markers including hs‐cTnT and NT‐proBNP, myocardial infarction history (including coronary surgery), cervical or lower limbs vessel plaque and medication. Clinical examinations were conducted by a trained staff group according to a standard guideline. In particular, height and weight were measured with patients standing without shoes and with lightweight clothing. Waist circumference was measured at the midpoint of the lowest rib and the iliac crest. BP was reported as the means of three consecutive measurements with an interval of 5 min. Body mass index (BMI) was calculated as weight divided by height squared (kg/m^2^).

### 2.3 Laboratory testing

Blood samples were obtained between 6:00 and 9:00 AM after fasting for at least 8 h. Venipuncture was performed in the median cubital vein, and centrifugation and dispensing were completed within 1 h. All samples were transported under a cold chain to a central laboratory for testing within 2–4 h. Sex hormones including total T (TT), E2, FSH, and LH were measured by electrochemiluminescence immunoassay (Roche E602, Roche, Basel, Switzerland). The minimal detectable limits for each hormone were as follows: 0.09 nmol/L (TT), 18.35 pmol/L (E2), and 0.1 IU/L (LH and FSH). Glycosylated hemoglobin (HbA1c) was assessed by high‐performance liquid chromatography (MQ‐2000PT, Medconn, Shanghai, China). Plasma glucose and lipid profiles were measured by Beckman Coulter AU 680 (Beckman Coulter, Krefeld, Germany). NT‐proBNP was measured in a central laboratory by using the Roche Elecsys assay (Roche Diagnostics). Concentrations of hs‐cTnT were determined using an hs‐cTnT assay (Roche Diagnostics GmbH) on a Modular Analytics E170 autoanalyzer (Roche Diagnostics). The lower detection limit (LOD) of the hs‐cTnT assay was 3 ng/L (according to the manufacturer's information). The 99th percentile upper reference limit (URL) for a reference population has been reported to be 14 ng/L^17^, ^18^, ^19^ and hs‐cTnT levels greater than 14 ng/L were considered elevated.

### 2.4 Outcomes of interest

The primary outcome was subclinical myocardial injury or necrosis defined by elevated hs‐cTnT. Subclinical myocardial injury or necrosis has been defined as hs‐cTnT ≥the 99th‐percentile URL (14 ng/L) in many recently published researches and this cutoff value has proven to be strongly associated with poorer adverse cardiovascular outcomes, and we thus followed these criteria to define subclinical myocardial injury.^17^, ^18^, ^19^ Elevated NT‐proBNP was another outcome of interest, and the cutoff value was 125 pg/mL.

### 2.5 Statistical analysis

The SPSS (Version 22.0) and R software (Version 3.6.1) were used to perform statistical analyses. Continuous variables were presented as median (interquartile range), while categorical variables were presented as frequency (%). Student's *t*‐test or Wilcoxon rank‐sum test was used for testing differences between groups for continuous variables, and chi‐square test was used for categorical variables. The correlations between LH level and variables related to cardiovascular risks were calculated with Pearson or Spearman's correlation test. To assess the correlations of hs‐cTnT with sex hormones including LH, FSH, E2, and T, curves were fitted using three models (linear model, locally weighted regression (LOESS) model and logarithmic model), and *R* ^2^ representing the coefficient of determination in the linear model was calculated. The correlations of LH and FSH with cardiac markers including hs‐cTnT and NT‐proBNP were further assessed by multiple linear regression to adjust for potential confounders, and results were expressed as standardized coefficients. Apart from cardiac injury or lesion, cTnT levels can also be influenced by factors such as skeletal muscle mass.^20^, ^21^ Consequently, we considered multiple potential confounders, including CK, into our model to refine our analysis. We also selected CRP as a potential confounder, as inflammatory cytokines have been reported to be closely related to serum LH level.^22^ The odds ratios (OR) and the corresponding 95% confidence intervals (CI) for elevated hs‐cTnT or elevated NT‐proBNP concerning each explanatory variable were obtained by logistic regression analysis. Biologically relevant variables with statistical significance were retained in the final multivariate model. All statistical analyses were two‐sided, and P values less than 0.05 were considered to be statistically significant.

## 3 RESULTS

### 3.1 Characteristics of participants

The clinical characteristics of 324 hospitalized postmenopausal female with T2DM are shown in Table 1. The median LH, FSH, E2, and T levels were 25.99 mIU/mL, 50.48 mIU/mL, 39.38 pmol/L and 0.64 nmol/L, respectively. Additionally, the median hs‐cTnT and NT‐proBNP levels respectively were 9 ng/L and 90.49 pg/mL.

**TABLE 1:** Clinical features of 324 postmenopausal T2DM patients.

Characteristics	*n* = 324
Age, years	70 (64–76)
BMI, kg/m^2^	25.7 (23.4–28.0)
DM duration, years	13 (6–20)
HbA1c, %	8.1 (7.0–9.4)
FPG, mmol/L	7.2 (6.0–9.0)
BP, mmHg	
Systolic	142 (128–156)
Diastolic	74 (67–81)
Sex hormones	
LH, mIU/mL	25.99 (19.41–32.32)
FSH, mIU/mL	50.48 (37.38–62.63)
E2, pmol/L	39.38 (21.99–66.98)
T, nmol/L	0.64 (0.39–0.89)
hs‐cTnT, ng/L	9 (7–13)
NT‐proBNP, pg/ml	90.49 (45.57–214.70)
Lipid profile, mmol/L	
LDL‐C	2.63 (1.96–3.28)
HDL‐C	1.27 (1.03–1.45)
TG	1.36 (0.99–2.03)
TC	4.47 (3.70–5.40)
mAlb, mg/L	7.05 (3.91–17.20)
CRP, mg/L	1.86 (0.96–3.51)
Smoking	5 (1.5%)
Drinking	3 (0.9%)

*Note*: Data are presented as the median (interquartile range) for continuous variables or percentage for categorical variables.

Abbreviations: BMI, body mass index; BP, blood pressure; CRP, C‐reactive protein; DM, diabetes mellitus; E2, estradiol; FPG, fasting plasma glucose; FSH, follicle‐stimulating hormone; HbA1c, glycated hemoglobin; HDL‐C, high‐density lipoprotein cholesterol; hs‐cTnT, high‐sensitive cardiac troponin T; LDL‐C, low‐density lipoprotein cholesterol; LH, luteinizing hormone; mAlb, microalbuminuria; NT‐proBNP, N‐terminal prohormone of brain natriuretic peptide; T, testosterone; T2DM, type 2 diabetes mellitus; TC, total cholesterol; TG, triglyceride.

### 3.2 Sex hormones and hs‐cTnT elevation

A total of 78 cases among the 324 postmenopausal T2DM patients were classified as having subclinical myocardial injury (hs‐cTnT ≥14 ng/L). Table 2 presents the differences in clinical variables between postmenopausal T2DM patients with and without subclinical myocardial injury. Compared to those without subclinical myocardial injury, postmenopausal T2DM patients with this condition exhibited higher LH (29.67 vs. 25.08 mIU/mL, *p* = 0.0001) and higher FSH (56.59 vs. 48.57 mIU/mL, *p* = 0.003), while there were no significant differences observed for E2 and T (Table 2). Postmenopausal T2DM patients with subclinical myocardial injury also had increased age (73 vs. 68 years, *p* < 0.0001), DM duration (20 vs. 10 years, *p* < 0.0001), systolic BP (SBP) (149 vs. 141 mmHg, *p* = 0.008) and CRP (2.48 vs. 1.67 mg/L, *p* = 0.033) (Table 2).

**TABLE 2:** Differences in the clinical variables between postmenopausal T2DM patients with and without subclinical myocardial injury (hs‐cTnT ≥14 ng/L).

Variables	Without subclinical myocardial injury (*n* = 246)	With subclinical myocardial injury (*n* = 78)	*p* value
Age, years	68 (63–75)	73 (68–79)	<0.0001*
DM duration, years	10 (5–19)	20 (10–24)	<0.0001*
Smoking, %	3 (%)	2 (%)	0.597
Drinking, %	2 (%)	1 (%)	0.564
BMI, kg/m^2^	25.7 (23.4–28.1)	25.6 (22.8–27.9)	0.749
HbA1c, %	8.0 (6.9–9.4)	8.4 (7.3–9.9)	0.135
BP, mmHg			
Systolic	141 (126–153)	149 (134–164)	0.008**
Diastolic	75 (68–83)	72 (64–79)	0.018***
Sex hormones			
LH, mIU/mL	29.67 (24.37–41.84)	25.08 (18.27–31.37)	0.0001*
FSH, mIU/mL	56.59 (43.66–70.93)	48.57 (35.84–61.91)	0.003**
E2, pmol/L	41.13 (26.52–62.62)	39.05 (21.39–68.26)	0.837
T, nmol/L	0.59 (0.35–0.95)	0.65 (0.41–0.87)	0.422
Lipid profile, mmol/L			
LDL‐C	2.66 (1.99–3.30)	2.47 (1.72–3.28)	0.431
HDL‐C	1.27 (1.03–1.45)	1.26 (1.02–1.44)	0.872
TG	1.38 (0.98–2.06)	1.28 (1.01–1.95)	0.339
TC	4.49 (3.7–5.44)	4.40 (3.67–5.28)	0.673
CRP, mg/L	1.67 (0.91–3.26)	2.48 (1.38–5.22)	0.033***
NT‐proBNP, pg/mL	75.67 (38.18–127.40)	241.60 (121.10–448.75)	<0.001*

*Note*: Data are presented as the median (interquartile range) for continuous variables or percentage for categorical variables.

Abbreviations: BMI, body mass index; BP, blood pressure; CRP, C‐reactive protein; DM, diabetes mellitus; E2, estradiol; FSH, follicle‐stimulating hormone; HbA1c, glycated hemoglobin; HDL‐C, high‐density lipoprotein cholesterol; hs‐cTnT, high‐sensitive cardiac troponin T; LDL‐C, low‐density lipoprotein cholesterol; LH, luteinizing hormone; NT‐proBNP, N‐terminal prohormone of brain natriuretic peptide; T, testosterone; T2DM, type 2 diabetes mellitus; TC, total cholesterol; TG, triglyceride.

*** *p* < 0.001;

** *p* < 0.01; *

**FIGURE 1:**
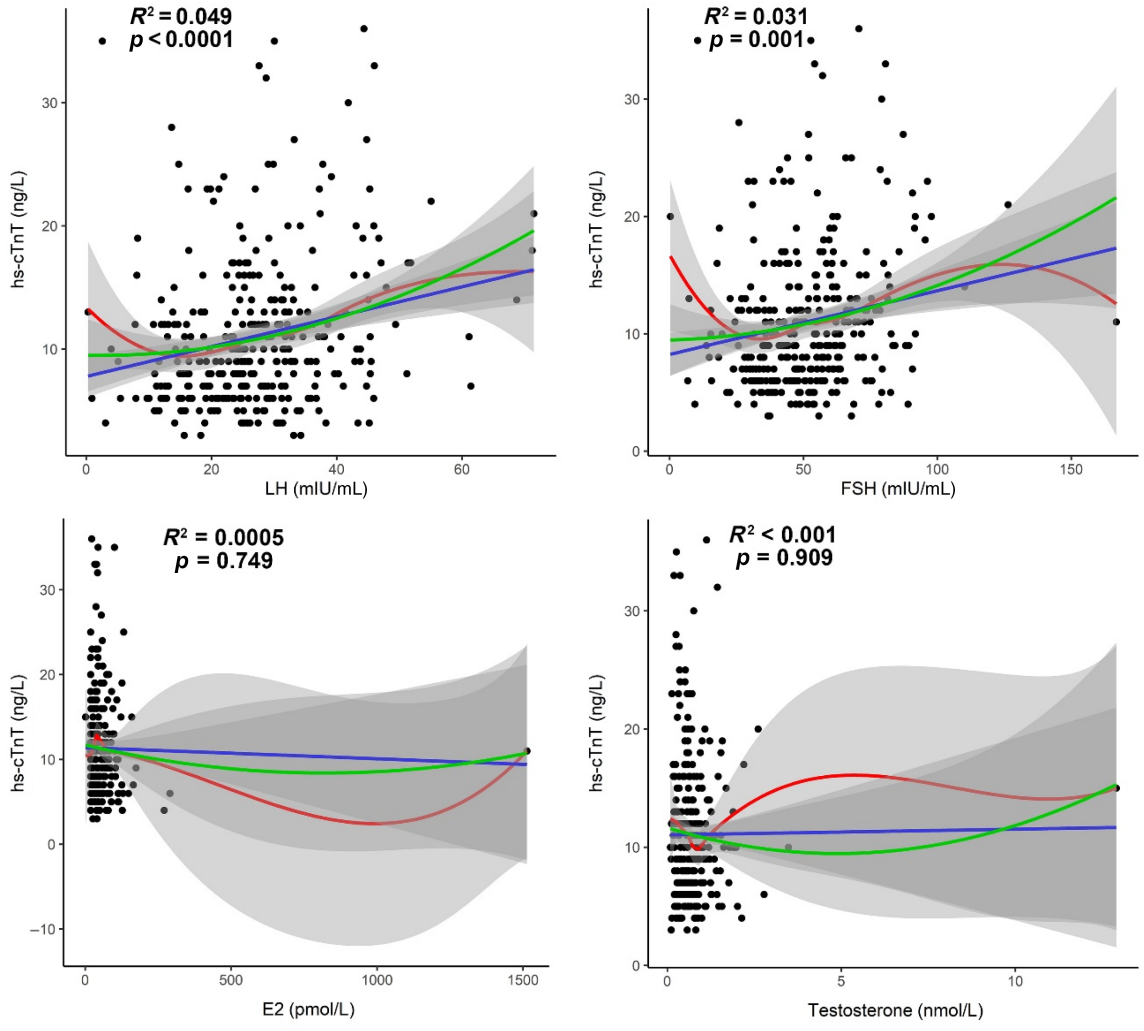
Correlations of high‐sensitive cardiac troponin T (hs‐cTnT) with luteinizing hormone (LH), follicle‐stimulating hormone (FSH), estradiol (E2) and testosterone (T) in postmenopausal type 2 diabetes mellitus (T2DM) patients. (Fitted curves of linear model are shown in blue; smoothing fitted curves of locally weighted regression (LOESS) model are in red; fitted curves of logarithmic model are in green. *R* ^2^ and *p* values from the linear model are shown in the figure.)

As shown in Figure 1, we analyzed the correlations between hs‐cTnT levels and sex hormones including LH, FSH, E2, and T among the 324 patients. LH exhibited a significantly positive correlation with hs‐cTnT (*R* ^2^ = 0.049, *p* < 0.0001), while FSH also demonstrated a significantly positive correlation with hs‐cTnT (*R* ^2^ = 0.031, *p* = 0.001). However, E2 and T were not significantly correlated with hs‐cTnT (*p* > 0.05; Figure 1).

Subsequently, we conducted further analyses to detect the correlations between LH, FSH and hs‐cTnT, after adjusting for potential confounders that are listed in Table 3. Multiple linear regression analyses revealed that LH was positively still correlated with hs‐cTnT (*β* = 0.189, *p* = 0.002) after adjusting for confounding factors. Considering the close relationship between LH levels and years after menopause, we conducted subgroup analysis by stratifying women into quartiles based on the years after menopause. The positive correlation between LH and hs‐cTnT persisted across all subgroups (Q1: *β* = 0.370, *p* = 0.015; Q2: *β* = 0.333, *p* = 0.005; Q3: *β* = 0.265, *p* = 0.046; Q4: *β* = 0.429, *p* = 0.016) (Table 4).

**TABLE 3:** Correlations of cardiovascular biomarkers with LH and FSH in postmenopausal T2DM patients.

Variable		LH (mIU/mL)		FSH (mIU/mL)	
		*β*	*p* value	*β*	*p* value
hs‐cTnT, ng/L	Univariate analysis	0.121	<0.001	0.054	0.001*
	Model 1^a^	0.166	<0.001	0.073	<0.001**
	Model 2^b^	0.178	0.002	−0.008	0.800
	Model 3^c^	0.189	0.002	−0.015	0.661
NT‐proBNP, pg/mL (Log2 transformed)	Univariate analysis	0.032	<0.001	0.013	0.008*
	Model 1^a^	0.033	0.001	0.015	0.015
	Model 2^b^	0.036	0.037	−0.003	0.802
	Model 3^c^	0.033	0.066	0.004	0.733
CRP, mg/L	Univariate analysis	−0.358	0.003	−0.254	<0.001**
	Model 1^a^	−0.119	0.182	−0.116	0.024***
	Model 2^b^	0.102	0.475	−0.162	0.051

Abbreviations: BMI, body mass index; BP, blood pressure; CK, creatine kinase; CRP, C‐reactive protein; DM, diabetes mellitus; E2, estradiol; FSH, follicle‐stimulating hormone; HbA1c, glycated hemoglobin; HDL‐C, high‐density lipoprotein cholesterol; hs‐cTnT, high‐sensitive cardiac troponin T; LDL‐C, low‐density lipoprotein cholesterol; LH, luteinizing hormone; NT‐proBNP, N‐terminal prohormone of brain natriuretic peptide; T, testosterone; T2DM, type 2 diabetes mellitus; TG, triglyceride.

^a^ Model 1: Adjustment for DM duration, BP, BMI, smoking, drinking, LDL‐C, HDL‐C, TG, CK, HbA1c, E2 and T.

^b^ Model 2: Further adjustment for either FSH or LH in addition to the variables in model 1.

^c^ Model 3: Further adjustment for CRP in addition to the variables in model 2.

*** *p* < 0.001;

** *p* < 0.01;

* *p* < 0.05.

**TABLE 4:** Correlations of hs‐cTnT with LH in postmenopausal T2DM patients stratified by quartiles of years after menopause.

Quartiles of years after menopause	Univariate		Multivariate^a^	
	*β*	*p* value	*β*	*p* value
Q1 (*N* = 79)	0.332	0.003*	0.370	0.015**
Q2 (*N* = 80)	0.238	0.034**	0.333	0.005*
Q3 (*N* = 81)	0.245	0.027**	0.265	0.046**
Q4 (*N* = 84)	0.312	0.004*	0.429	0.016**

*Note*: The quartiles for stratification based on years after menopause were defined as follows: Q1: 0–14 years, Q2: 14–19 years, Q3: 20–25 years, Q4 ≥ 26 years.

Abbreviations: BMI, body mass index; BP, blood pressure; CK, creatine kinase; CRP, C‐reactive protein; E2, estradiol; FSH, follicle‐stimulating hormone; HbA1c, glycated hemoglobin; HDL‐C, high‐density lipoprotein cholesterol; hs‐cTnT, high‐sensitive cardiac troponin T; LDL‐C, low‐density lipoprotein cholesterol; LH, luteinizing hormone; T, testosterone; T2DM, type 2 diabetes mellitus; TG, triglyceride.

^a^ Adjustment for BP, BMI, smoking, drinking, LDL‐C, HDL‐C, TG, CK, HbA1c, E2, T, FSH, and CRP.

*** *p* < 0.001;

** *p* < 0.01;

* *p* < 0.05.

The outcomes of the logistic regression analysis were shown in Table 5. An independent and significant association between elevated LH and subclinical myocardial injury in postmenopausal T2DM patients was confirmed by multivariate logistic regression analysis (Adjusted OR = 1.077, 95% CI = 1.033–1.124; *p* < 0.001). However, there was no independent association observed between other gonadotropins, including FSH, and subclinical myocardial injury in postmenopausal T2DM patients (*p* > 0.05). Moreover, we explored the associations between hs‐cTnT elevation and several anthropometric/metabolic factors except for sex hormones. As shown in Table 5, DM duration and TG were independently associated with subclinical myocardial injury in postmenopausal T2DM patients (*p* < 0.05).

**TABLE 5:** Logistic regression analysis of the association between hs‐cTnT elevation and several anthropometric/metabolic factors.

Variable	Univariate		Multivariate^a^	
	OR (95%CI)	*p* value	OR (95%CI)	*p* value
BMI, kg/m^2^	1.004 (0.935–1.079)	0.903	—	—
HbA1c, %	1.115 (0.976–1.274)	0.109	—	—
DM duration, years	1.073 (1.041–1.106)	<0.001***	1.071(1.037–1.106)	<0.001***
CRP, mg/L	1.001 (0.989–1.013)	0.894	—	—
LDL‐C (mmol/L)	0.966 (0.746–1.251)	0.794	—	—
HDL‐C (mmol/L)	0.963 (0.433–2.138)	0.925	—	—
TG (mmol/L)	0.757 (0.558–1.025)	0.072	0.580(0.365–0.920)	0.021*
CK (U/L)	1.000 (0.975–1.026)	0.974	—	—
LH, mIU/mL	1.052 (1.027–1.077)	<0.001***	1.077(1.033–1.124)	<0.001***
FSH, mIU/mL	1.020 (1.007–1.033)	0.002**	0.988(0.966—1.011)	0.312
E2, pmol/L	0.998 (0.992–1.004)	0.542	—	—
T, nmol/L	1.178 (0.873–1.592)	0.284	—	—
Smoking	2.132 (0.350–12.994)	0.412	—	—
Drinking	1.584 (0.142–17.713)	0.709	—	—

Abbreviations: BMI, body mass index; CK, creatine kinase; CRP, C‐reactive protein; DM, diabetes mellitus; E2, estradiol; FSH, follicle‐stimulating hormone; HbA1c, glycated hemoglobin; HDL‐C, high‐density lipoprotein cholesterol; LDL‐C, low density lipoprotein cholesterol; LH, luteinizing hormone; T, testosterone; TG, triglyceride.

^a^ Adjustment for variables with *p* value less than 0.10 in the univariate analyses.

* Means *p* < 0.05;

** Means *p* < 0.01;

*** Means *p* < 0.001.

### 3.3 Sex hormones and elevated NT‐proBNP

As NT‐proBNP is known as another common cardiovascular biomarker, and elevated NT‐proBNP (>125 pg/mL) has been recognized as a predictor of heart failure, we then investigated the risk factors of elevated NT‐proBNP among 298 subjects with the measurement of NT‐proBNP. Based on the level of NT‐proBNP (cutoff value: 125 pg/mL), 298 patients were divided into two groups: 115 patients with elevated NT‐proBNP, and 183 patients with normal NT‐proBNP (Table 6). Differences in the clinical variables between postmenopausal T2DM patients with and without elevated NT‐proBNP (>125 pg/mL) are shown in Table 6. While there were no significant differences in BMI and HbA1c between the two groups, patients with elevated NT‐proBNP tended to be older and experience a longer T2DM duration (Table 6). Compared with those without elevated NT‐proBNP, postmenopausal T2DM patients with elevated NT‐proBNP did not have statistically significant changes in those gonadotropins including LH, FSH, E2, and T (*p* > 0.05; Table 6).

**TABLE 6:** Differences in the clinical variables between postmenopausal T2DM patients with and without elevated NT‐proBNP.

Variables	Elevated NT‐proBNP (*n* = 115)	Normal NT‐proBNP (*n* = 183)	*p* value
Age, years	73 (68–78)	68 (62–74)	<0.001*
DM duration, years	17 (8–20)	11 (5–20)	0.002**
Smoking, %	1 (0.9%)	4 (2.2%)	0.652
Drinking, %	0 (0%)	3 (1.6%)	0.287
BMI, kg/m^2^	25.4 (23.0–28.3)	25.6 (23.4–27.7)	0.727
HbA1c, %	8.1 (7.0–9.9)	8.1 (6.9–9.4)	0.571
BP, mmHg			
Systolic	148 (136–168)	138 (124–151)	<0.001*
Diastolic	73 (65–80)	74 (67–82)	0.248
Sex hormones			
LH, mIU/mL	27.10 (19.94–35.35)	25.29 (18.93–31.67)	0.111
FSH, mIU/mL	54.79 (38.6–64.96)	48.52 (36.28–61.25)	0.166
E2, pmol/L	43.92 (27.26–66.98)	43.92 (27.26–66.98)	0.088
T, nmol/L	0.66 (0.38–1.03)	0.61 (0.37–0.84)	0.313
Lipid profile, mmol/L			
LDL‐C	2.39 (1.81–3.20)	2.67 (2.02–3.29)	0.072
HDL‐C	1.25 (0.98–1.45)	1.29 (1.06–1.44)	0.571
TG	1.25 (0.93–1.82)	1.54 (1.03–2.17)	0.011***
TC	4.30 (3.64–5.11)	4.54 (3.74–5.47)	0.192
CRP, mg/L	2.12 (1.16–5.44)	1.88 (0.92–3.10)	0.064
hs‐cTnT, ng/L	13 (10–19)	8 (6–11)	<0.001*

*Note*: Data are presented as the median (interquartile range) for continuous variables or percentage for categorical variables.

Abbreviations: BMI, body mass index; BP, blood pressure; CRP, C‐reactive protein; DM, diabetes mellitus; E2, estradiol; FSH, follicle‐stimulating hormone; HbA1c, glycated hemoglobin; HDL‐C, high‐density lipoprotein cholesterol; hs‐cTnT, high‐sensitive cardiac troponin T; LDL‐C, low‐density lipoprotein cholesterol; LH, luteinizing hormone; NT‐proBNP, N‐terminal prohormone of brain natriuretic peptide; T, testosterone; T2DM, type 2 diabetes mellitus; TC, total cholesterol; TG, triglyceride.

*** *p* < 0.001;

** *p* < 0.01;

* *p* < 0.05.

According to Table 3, although LH and FSH were correlated with Log2‐transformed NT‐proBNP in the linear regression analysis, the correlations became insignificant after adjusting for CRP and other confounders. In addition, none of those sex hormones including LH, FSH, E2, and T were significantly associated with elevated NT‐proBNP in the logistic regression analysis (*p* > 0.05; Table 7). Among other anthropometric/metabolic factors, DM duration and CRP were independently associated with elevated NT‐proBNP in postmenopausal T2DM patients (*p* < 0.05; Table 7).

**TABLE 7:** Factors associated with elevated NT‐proBNP in postmenopausal T2DM patients through logistic regression analysis.

Variable	Univariate		Multivariate^a^	
	OR (95% CI)	*p* value	OR (95% CI)	*p* value
BMI, kg/m^2^	1.021 (0.957–1.089)	0.531	—	—
HbA1c, %	1.032 (0.912–1.167)	0.621	—	—
DM duration, years	1.044 (1.016–1.073)	0.002**	1.044 (1.016–1.073)	0.002**
CRP, mg/L	1.016 (1.000–1.031)	0.043*	1.019 (1.002–1.036)	0.027*
LDL‐C (mmol/L)	0.851 (0.669–1.082)	0.187	—	—
HDL‐C (mmol/L)	0.826 (0.402–1.695)	0.602	—	—
TG (mmol/L)	0.804 (0.627–1.032)	0.086	—	—
CK (U/L)	0.988 (0.963–1.013)	0.344	—	—
LH, mIU/mL	1.017 (0.996–1.038)	0.109	—	—
FSH, mIU/mL	1.007 (0.995–1.019)	0.250	—	—
E2, pmol/L	0.999 (0.997–1.002)	0.813	—	—
T, nmol/L	1.413 (0.882–2.264)	0.151	—	—
Smoking	0.393 (0.043–3.556)	0.406	—	—

Abbreviations: BMI, body mass index; CRP, C‐reactive protein; CK, creatine kinase; DM, diabetes mellitus; E2, estradiol; FSH, follicle‐stimulating hormone; HbA1c, glycated hemoglobin; HDL‐C, high‐density lipoprotein cholesterol; LDL‐C, Low density lipoprotein cholesterol; LH, luteinizing hormone; TG, triglyceride; T, testosterone.

^a^ Adjustment for variables with *p* value less than 0.10 in the univariate analyses.

* *p* < 0.05.

** *p* < 0.01.

As CRP was correlated with both hs‐cTnT and NT‐proBNP (Table 2 and Table 7), we consider CRP as an important confounder in the correlation between sex hormones and cardiac biomarkers.

## 4 DISCUSSION

In this cross‐sectional study, we analyzed anthropometric and laboratory data from 324 hospitalized postmenopausal T2DM patients and emphasized the associations between gonadotropins and biomarkers of ischemic heart diseases. We found a positive correlation between LH and elevated hs‐cTnT, which was independent of common CVD risk factors, such as age, DM duration, BMI, BP, smoking, lipid profile, CRP, E2, T, and FSH.

Hs‐cTnT is known as a marker of ischemic myocardial damage and serves as an accessible method for assessing subclinical myocardial injury or subclinical myocardial necrosis (SMN).^23^ SMN is characterized by the reduced vasodilatory capacity of small vessels and impaired nutritive capillary circulation in diabetic patients, the development of which mainly involves microvascular lesions such as transient microvascular ischemic episodes, microembolism, or small‐vessel occlusion.^24^, ^25^, ^26^ Studies on the skin microcirculatory reactivity also confirmed the potential utility of peripheral microcirculatory variables in the assessment of myocardial ischemia, providing further evidence of the involvement of microvascular impairment in the mechanisms underlying SMN. In addition, heightened inflammatory activation and reduced anti‐oxidative process contribute to the development of SMN.^27^, ^28^

The results of our study suggest a positive correlation between LH and SMN. Previous studies have reported the associations between LH and diabetic microvascular diseases. For example, one recent study identified LH as an independent risk factor for diabetic retinopathy in men, and another determined it as an independent predictor of diabetic kidney disease in men and postmenopausal women, while the relationship between LH and myocardial microvascular ischemia have not been explored in previous studies.^29^

Although LH receptor has been discovered in multiple tissues including heart muscle, the direct effect of LH on cardiovascular systems via receptor has not been reported. Here, we have summarized four potential explanations for the association between LH and SMN: (1) Elevated LH levels are associated with heightened inflammatory activation. Increased LH has been shown to correlate with elevated levels of several key proinflammatory cytokines in serum such as tumor necrosis factor (TNF)‐α, interleukin‐2 (IL‐2), IL‐2 receptor (IL‐2R), IL‐8, monocyte chemoattractant protein‐1, macrophage inflammatory protein‐1, and eotaxin. This signifies a proinflammatory state.^22^ Inflammatory processes and proinflammatory state could render an increase in vascular permeability, endothelial cell apoptosis, and chronic inflammation, which lead to impaired coronary microcirculation and reduced maximal blood flow in myocardial microvessels, serving as one of the mechanisms of SMN.^24^, ^30^, ^31^ (2) Elevated LH stimulates vascular endothelial growth factor (VEGF) expression, which is linked to microvascular dysfunction. VEGF is known to promote angiogenesis, increase vascular permeability, and facilitate cell migration and is often associated with poor prognosis and disease severity in several ASCVDs.^32^ In diabetic patients, VEGF plays an important role in endothelial dysfunction, which leads to diabetic microvascular complications.^24^ The angiogenic effect of VEGF could promote the growth and vulnerability of plaques, potentially leading to the influx of inflammatory cells and microembolism within vulnerable plaques.^33^ Moreover, one recent study found that plasma VEGF accumulation was associated with an increased T‐helper 1 (Th1)/T‐helper 2 (Th2) ratio in patients with T2DM, and the Th1/Th2 ratio was significantly higher in T2DM patients with microvascular complications than those without microvascular complications, suggesting that immune imbalance may also mediate the association between VEGF and microvascular dysfunction in diabetic patients.^24^ LH, as a proangiogenic hormone, has been reported to up‐regulate VEGF levels in several organs aside from ovaries, including eyes and kidneys, where the LH receptor has also been found.^34^, ^35^, ^36^, ^37^, ^38^ Therefore, we hold the view that elevated LH may lead to an increase in circulating VEGF level and the subsequent VEGF accumulation may contribute to endothelial dysfunction and destabilization of plaques, thus promoting the development of SMN. (3) Elevated LH is linked to increased oxidative processes. Before menopause, LH surge is known to initiate a massive recruitment of oxygen species, which modulates oocyte development and function.^39^ Aside from ovaries, elevation in LH was also found to parallel oxidative markers in research on neuronal degeneration.^40^ As increased oxidation is another mechanism for SMN, the link between LH and oxidative processes could partly explain the elevation in hs‐cTnT. However, further evidence is required to support the association between elevated LH and vascular oxidative stress. (4) Elevated LH attenuates vasodilation, resulting in reduced blood flow in small vessels. As has been mentioned above, reduced vasodilatory capacity of small vessels is one major characteristic of SMN in diabetic patients. In ovariectomized Apolipoprotein E^−/−^ female mice, high levels of LH have been shown to attenuate endothelial nitric oxide (NO)‐dependent vasodilation by inhibiting NO synthesis via the phosphatidylinositol 3‐kinase/protein kinase B signaling pathway.^41^ Reduced vasodilatory capacity may lead to ischemic episodes of small vessels, which could be one of the mechanisms contributing to SMN.

Although SMN is mainly a reflection of myocardial microvascular function, mounting evidence indicates an association between abnormal microcirculation and risks of adverse cardiac events. Previous studies indicated that the presence of microvascular diseases in diabetic patients not only signified a severe condition of ischemic cardiomyopathy but also contributed to the development of cardiovascular and cerebrovascular events.^42^, ^43^ For example, microvascular disease, rather than macrovascular disease, may be a risk factor for ischemic stroke in diabetic patients.^44^, ^45^ In fact, some researches have determined hs‐cTnT as a predictor of major adverse cardiac events in stable cardiac patients and diabetic patients even being well below the diagnostic range defined by consensus.^26^, ^46^, ^47^ These indications underline the prognostic value of our study and indicate that the assessment of LH level can help stratify the potential risks of adverse cardiac events in postmenopausal T2DM female patients to guide secondary preventive efforts.^46^ Nonetheless, further research is necessary to uncover the mechanisms of association between LH and myocardial microvascular dysfunction and risk factors of atherosclerosis respectively to comprehensively understand the role of LH in promoting the progression of ischemic heart disease.

Elevated NT‐proBNP (>125 pg/mL) is a strong predictor of a high risk of heart failure in diabetic patients.^48^ Previous studies also found that older age and longer DM duration were related to higher risks of heart failure.^49^, ^50^ However, there is a dearth of research addressing the role of LH in the development of heart failure, with only one study reporting a positive correlation between gonadotropins levels and QT interval duration.^51^ Thus, the roles of LH in cardiac insufficiency remain to be clarified. Higher levels of LH may be key factors that predict a relatively high risk of heart failure for postmenopausal patients with T2DM, which needs to be explored in additional studies with a larger sample size.

There are several limitations of our study. First, as this is a cross‐sectional design, we cannot infer the causal relationships between LH and hs‐cTnT or multiple cardiovascular risk factors. Second, the sample size of subjects with cardiac marker measurements is relatively small, and all subjects in this study were enrolled from Shandong Province, so the findings cannot generalize to other regions of China. Third, there is a need for experimental evidence to show whether microcirculation‐related biological mechanisms can explain the association between LH and hs‐cTnT.

In summary, our data suggest that in postmenopausal T2DM patients, elevated LH levels were positively and independently associated with increased hs‐cTnT levels, which indicate the severity degree of subclinical myocardial injury. Our findings indicate that elevated LH may be linked with myocardial microvascular dysfunction in T2DM patients, and LH levels may help stratify potential risks of adverse cardiac events to guide secondary preventive efforts.

## AUTHOR CONTRIBUTIONS

Yahao Wang performed statistical analysis, interpreted the results of experiments, and drafted the paper. Yixuan Li collected the clinical data and assisted in the data analysis and manuscript drafting. Chuanfeng Liu assisted with the statistical analysis and provided helpful suggestions. Yiming Li and Yangang Wang conceived and designed the study. Yiming Li supervised the project.

## FUNDING INFORMATION

This study had no funding support.

## CONFLICT OF INTEREST STATEMENT

The authors declare no conflicts of interest.

## Data Availability

The data used to support the findings of this study were selected from the database of inpatients at the Affiliated Hospital of Medical College Qingdao University (Shandong, China) and are available from the corresponding author upon request.

## Footnotes

*
*p* < 0.05.
